# Identification of Neurotransmission and Synaptic Biological Processes Disrupted in Autism Spectrum Disorder Using Interaction Networks and Community Detection Analysis

**DOI:** 10.3390/biomedicines11112971

**Published:** 2023-11-04

**Authors:** Joana Vilela, Hugo Martiniano, Ana Rita Marques, João Xavier Santos, Muhammad Asif, Célia Rasga, Guiomar Oliveira, Astrid Moura Vicente

**Affiliations:** 1 Departamento de Promoção da Saúde e Doenças Não Transmissíveis, Instituto Nacional de Saúde Doutor Ricardo Jorge, Avenida Padre Cruz, 1649-016 Lisboa, Portugal; joana.vilela@insa.min-saude.pt (J.V.); hugo.martiniano@insa.min-saude.pt (H.M.); a.rita.marques@insa.min-saude.pt (A.R.M.); joao.xavier@insa.min-saude.pt (J.X.S.); muhasif123@gmail.com (M.A.); celia.rasga@insa.min-saude.pt (C.R.); 2 BioISI-Biosystems & Integrative Sciences Institute, Faculty of Sciences, University of Lisboa, Campo Grande, C8, 1749-016 Lisboa, Portugal; 3 Department of Bioinformatics and Biotechnology, Government College University Faisalabad, Faisalabad 38000, Pakistan; 4 Unidade de Neurodesenvolvimento e Autismo, Serviço do Centro de Desenvolvimento da Criança, Centro de Investigação e Formação Clínica, Hospital Pediátrico, Centro Hospitalar e Universitário de Coimbra (CHUC), 3000-602 Coimbra, Portugal; guiomar@chuc.min-saude.pt; 5 Coimbra Institute for Biomedical Imaging and Translational Research, University Clinic of Pediatrics, Faculty of Medicine, University of Coimbra, 3000-602 Coimbra, Portugal

**Keywords:** autism spectrum disorder, ultra-rare variants, protein–protein interaction analysis, synaptic and neurotransmitter genes, community detection analysis

## Abstract

Autism Spectrum Disorder (ASD) is a neurodevelopmental disorder characterized by communication deficits and repetitive behavioral patterns. Hundreds of candidate genes have been implicated in ASD, including neurotransmission and synaptic (NS) genes; however, the genetic architecture of this disease is far from clear. In this study, we seek to clarify the biological processes affected by NS gene variants identified in individuals with ASD and the global networks that link those processes together. For a curated list of 1216 NS candidate genes, identified in multiple databases and the literature, we searched for ultra-rare (UR) loss-of-function (LoF) variants in the whole-exome sequencing dataset from the Autism Sequencing Consortium (N = 3938 cases). Filtering for population frequency was carried out using gnomAD (N = 60,146 controls). NS genes with UR LoF variants were used to construct a network of protein–protein interactions, and the network’s biological communities were identified by applying the Leiden algorithm. We further explored the expression enrichment of network genes in specific brain regions. We identified 356 variants in 208 genes, with a preponderance of UR LoF variants in the *PDE11A* and *SYTL3* genes. Expression enrichment analysis highlighted several subcortical structures, particularly the basal ganglia. The interaction network defined seven network communities, clustering synaptic and neurotransmitter pathways with several ubiquitous processes that occur in multiple organs and systems. This approach also uncovered biological pathways that are not usually associated with ASD, such as brain cytochromes P450 and brain mitochondrial metabolism. Overall, the community analysis suggests that ASD involves the disruption of synaptic and neurotransmitter pathways but also ubiquitous, but less frequently implicated, biological processes.

## 1. Introduction

Autism Spectrum Disorder (ASD) is a neurodevelopmental disorder characterized by communication deficits and repetitive behavioral patterns [1]. Individuals with ASD may present a variety of clinical and behavioral symptoms, as well as a number of comorbidities, leading to variable degrees of clinical severity. Patients with ASD may range from autonomous individuals with typical or above-average cognitive levels to individuals with moderate to severe Intellectual Disability (ID) and/or other comorbidities who need support to perform even basic daily tasks. ASD is known to segregate in families [2,3,4], and genetics has a significant contribution to the disease’s development. ASD occurs three to four times more frequently in males than females, but the origin of this skewed sex ratio is not well established. While genetic factors protective in females may be implicated, some authors also suggest that it may reflect diagnostic biases [5]. Up to 40% of the patients have an identifiable single gene disorder or chromosomal alteration such as Fragile X syndrome caused by *FMR1* gene expansions; Rett syndrome, which results from *MECP2* mutations; or Angelman or Prader-Willi syndromes, involving 15q11-q13 deletions [6]; however, for most patients, the etiology is unknown, and the overall genetic architecture underlying the disease remains unclear. There is evidence supporting the involvement of a large number of risk genes [7,8,9]. Massive efforts to understand the genetics of ASD indicate that no single neurobiology underlies this disorder [10,11,12] and there is a strong heterogeneity in the molecular mechanisms and biological processes that can be affected, likely reflecting the spectrum of clinical phenotypes [13]. Genes encoding proteins implicated in chromatin remodeling, regulation of transcription, cell proliferation and synaptic mechanisms [8,14] have been commonly associated with ASD. In particular, there is strong genomic and functional evidence indicating that neurotransmission and synaptic biological processes are altered in ASD [13,15,16,17]. The identification of the molecular processes that drive synaptic dysfunction in ASD and the biological pathways involved remains an important question.

Neurotransmitters such as gamma-aminobutyric acid (GABA), glutamate, serotonin or dopamine play a fundamental role in brain development and contribute to memory formation, cognition, behavior and motor activity [18]. Neurotransmitter system dysfunctions lead to impairments in brain development that have been implicated in ASD and involve key processes such as neuronal cell migration, differentiation, synaptogenesis, apoptosis and synaptic pruning [13,19]. The synapse has been extensively studied in ASD since the identification of ASD mutations in neuroligin genes [20,21], which encode synaptic adhesion molecules. There is strong evidence that genes encoding synaptic proteins, including synaptic adhesion molecules such as neuroligins, neurexins, cadherins and contactins, synaptic scaffold proteins, ion channels and neurotransmitter receptors are involved in ASD and in other neurodevelopmental and neurological disorders [22]. Many ASD candidate genes are also involved in the formation of synaptic structures such as dendritic spines, which have shown abnormalities in terms of number and shape that lead to brain dysfunction in ASD and several neurological disorders [23]. In addition, mutations in the pre-synaptic genes *RIMS3/NIM3* and the post-synaptic genes *IL1RAPL1* and *SYNGAP1*, involving synaptic vesicle organization or synapse formation, have also been associated with ASD [24,25].

Alterations in gabaminergic and glutaminergic systems cause excitatory/inhibitory imbalances and are potential mechanisms for ASD symptomatology and for several other neurodevelopmental, neurologic and neuropsychiatric disorders such as global developmental delay, intellectual disability, epilepsy or schizophrenia [19]. Excitatory/inhibitory neurotransmission imbalance is often reported in ASD [26] and is thought to be due to abnormal glutamatergic excitatory and GABAergic inhibitory neurotransmission in several brain regions, affecting information processing and behavior [26]. Cortical excitatory/inhibitory imbalance could explain social and cognitive deficits [27] and may result from alterations in initial neural circuit formation or maintenance since several of the genes derived from linkage and association studies encode proteins involved in these processes [28,29]. Postmortem studies have also revealed structural and/or functional alterations in glutamatergic and GABAergic pathways in ASD [30,31].

Hundreds of ASD putative risk genes have been reported in the last years [8,32,33], and it is expected that mutations in each of these genes contribute to a small proportion of ASD risk. A large fraction of the ASD risk variance is attributed to common variants of small effect [34,35], which when combined in a particular individual reach the threshold for disease expression, with heterogenous clinical presentation; however, large-effect rare and ultra-rare (UR) variants have important contributions to ASD risk and deserve extensive analysis. A recent study suggested that inherited UR likely gene disruptive (LGD) variants contribute to at least 4.5% of ASD risk [36]. Previous studies showed that the transmission of UR LGD variants contributes to ASD diagnosis in 5.4% of the families analyzed [37] and private, inherited LGD Single-Nucleotide Variants (SNVs) contribute to 8.5% of ASD risk in the population analyzed [38]. 

Our main aim in this study was to further understand the role of neurotransmission and synaptic (NS) mechanisms in ASD. Therefore, we sought to identify communities of protein–protein interactions involved in ASD, defined by variants in NS genes found in large datasets of ASD subjects. For this, we carried out a systematic analysis of public databases and identified the biological pathways and protein communities affected by these genes. We specifically focused on UR variants in NS genes as these variants more likely constitute large-effect risk variation with potential for the development of personalized medicinal interventions.

## 2. Materials and Methods

### 2.1. Generation of a List of Neurotransmission and Synaptic ASD Candidate Genes

To explore the involvement of specific synaptic and neurotransmission pathways in ASD, we selected a comprehensive list of NS genes that may underlie the disease etiology and help establish a biological disease model. The focus was on selecting genes that are relevant for neurotransmission and synaptic processes since there is evidence that these mechanisms are affected in ASD. The gene search was conducted by querying different databases that combine information from several biological processes and the genes involved in those mechanisms, namely, Gene Ontology (GO) [39], KEGG pathway database [40,41,42], Reactome [43], SynaptomeDB [44] and SynSysNet [45] (see workflow in Supplementary Table S1a). The Gene Ontology (GO) resource (http://geneontology.org/ (accessed on 14 September 2018)) develops structured controlled ontologies to characterize genes and their products. The GO Consortium has developed AmiGO, a web-based application that allows users to search, sort, analyze, visualize and download data of interest. The KEGG Pathway (https://www.genome.jp/kegg/pathway.html (accessed on 14 September 2018)) is a collection of manually drawn biological pathway maps displaying current knowledge on the molecular interactions and reactions, and the relations with diverse biological networks. Reactome (https://reactome.org/ (accessed on 20 September 2018)) is a manually curated and peer-reviewed pathway database that provides bioinformatics tools for the visualization, interpretation and analysis of pathway knowledge with applications in genome analysis, modeling and systems biology. SynaptomeDB (http://metamoodics.org/SynaptomeDB/index.php (accessed on 26 September 2018)) and SynSysNet (http://bioinformatics.charite.de/synsys/index.php?site=home (accessed on 26 September 2018)) are databases specifically for synaptic genes and proteins. SynSysNet is a European expertise network, building a synapse molecular interactome, and provides a highly curated online database of synaptic proteins. SynaptomeDB is an integrated database to retrieve, compile and annotate genes comprising the synaptome. SynaptomeDB genes encode components of the synapse as neurotransmitters and their receptors, adhesion/cytoskeletal proteins, scaffold proteins or transporters. For NS candidate gene identification, we selected all the genes with GO annotations for the terms “neurotransmitter” and “synapse” with experimental evidence from *Homo sapiens* studies, and the Reactome genes included in the “Neuronal System” pathway. We also selected genes from KEGG pathways that are synapse-specific and from other pathways that occur in several body systems, selecting the annotations for “neurotransmitter” and “synapse” in *Homo sapiens*. We selected genes from the following KEGG pathways: Dopaminergic; Glutamatergic; GABAergic; Cholinergic; Serotonergic; Retrograde Endocannabinoid signaling; Long-term depression; Synaptic vesicle cycle; Long-term potentiation; Neurotrophin signaling; Cocaine addiction; Nicotine addiction; Morphine addiction; Amphetamine addiction; Alcoholism; cAMP; Calcium signaling; PI3K-AKT; and Cell Adhesion Molecules (CAMs). SynaptomeDB and SynSysNet databases were used to help in the identification of NS genes in KEGG pathways that are present in several body systems. Finally, the Simons Foundation Autism Research Initiative (SFARI) database [46] gene scoring module was consulted and a literature review on PUBMED of the publications available on genes related with “ASD” and/or “autism” was conducted to provide additional evidence to the pre-selected genes. 

### 2.2. Datasets Analysed in This Study

In this study, we analyzed three datasets in different stages of the analyses. We used the Autism Sequencing Consortium (ASC) exome dataset to analyze SNVs in 3938 individuals with ASD and 1124 unaffected controls [47] (dbGaP accession phs000298.v1.p1; phs000298.v2.p2; phs000298.v3.p2; phs000298.v4.p3). For filtering SNVs by population frequency, we used the control dataset from the Genome Aggregation database (gnomAD v2.1.1), which incorporates the exome information from 60,146 controls sequenced as part of various disease-specific and population genetic studies [48]. The gnomAD dataset was developed to provide large-scale data of genetic variation present in several populations, to filter out common benign/neutral variants and identify variants with clinical meaning based on the frequency in the human population. Additionally, we analyzed a dataset of de novo SNVs, as variants of this class are rare and can have a strong effect in protein function or structure, constituting important targets for the discovery of ASD risk genes [49,50]. For this identification, we used the Simons Simplex Collection (SSC) de novo dataset, which includes a number of ASD whole-exome, whole-genome and targeted sequencing studies, comprising a total of 11,905 ASD cases and 7265 controls. Supplementary Table S1b contains a list of all of these studies. For the Copy Number Variants (CNVs) identification, we analyzed genetic data from the Autism Genome Project (AGP) consortium (N = 2446) (accession code: phs000267.v5.p2) [51,52]. The AGP is an international consortium with over 50 sites in North America and Europe, and the dataset is composed of samples of trio families comprising an affected proband and two parents [51,53,54,55]. 

### 2.3. Identification of Ultra-Rare Loss-of-Function SNVs Targeting Neurotransmission and Synaptic Genes in ASD Exomic Datasets

The identification of NS genes targeted by UR (minor allele frequency (MAF)  <  0.1%) SNVs was carried out in the ASC Whole Exome Sequencing (WES) dataset of subjects with ASD [47]. For this purpose, we searched for SNVs in genes of the candidate list previously defined. Quality control filters applied to exome data (using the parameters defined in bcftools; https://samtools.github.io/bcftools/ (accessed on 15 November 2018) were as follows: Variant Quality Score, VSQLOD ≥ −1.5; Read Depth, DP > 8; Genotype Quality, GQ > 20; Allelic Depth, AD > 0.2; and Missingness < 10%). For the selection of pathogenic variants, we analyzed the WES resulting Variant Call Format (VCF) files in the ASC ASD cohort. We filtered out the variants that were present in the control datasets included in the gnomAD [48] (http://gnomad.broadinstitute.org/ (accessed on 15 November 2018)) with MAF ≥ 0.1%, keeping only the UR variants for further analyses; inspected the variants with MAF < 0.1% in cases and controls; and predicted pathogenic impact in protein function or structure. Our focus is on UR variants, which are expected to have a high effect on the phenotype. We selected loss-of-function (LoF) variants (variants of frameshift, stop gain, start lost, splice acceptor and splice donor) according to the Ensembl Variant Effect Predictor (VEP) [56].

### 2.4. Construction of the Protein–Protein Interaction Network Spanned by Neurotransmission and Synaptic Genes Affected in ASD Probands

In this step, we constructed a protein–protein interaction (PPI) network of proteins encoded by the genes targeted by UR pathogenic SNVs using the STRING database (version 11.0; http://string-db.org/ (accessed on 3 January 2019)) [57]. STRING comprises multiple datasets such as genomic data, co-expression or results from high-throughput experiments, which are used to infer associations among proteins, including physical and functional parameters. Protein–protein interaction networks are mathematical representations of the physical contacts between proteins in the cell. These contacts are specific, occur between defined binding regions in the proteins and have a particular biological meaning. The workflow designed for this task is shown in Supplementary Figure S1a. The list of genes affected by UR LoF SNVs was used as input for STRING network analyses. A multiple-protein analysis was conducted using the edge weights, and a network representation was designed using Cytoscape v3.8.2. [58].

### 2.5. Protein–Protein Interaction Network Community Detection

We are interested in identifying the biological processes affected in the network that are common to a larger group of patients instead of analyzing single genes and individual alterations. To do this, we identified the network biological communities by applying the Leiden community detection algorithm [59] implemented in the CDlib python package (https://cdlib.readthedocs.io/en/latest/reference/cd_algorithms/algs/cdlib.algorithms.leiden.html#cdlib.algorithms.leiden (accessed on 16 January 2019)) [60], based on modularity optimization to depict partitions and the hierarchical community structure. Using this method with the default parameters, we decomposed the network into subunits or communities, which are sets of densely connected nodes. Nodes belonging to different communities are only sparsely connected. Network summary statistics were calculated for the parameters related with counts of nodes and edges, node degree and average local clustering coefficient. The identification of network functional protein communities (biological communities) may uncover a priori unknown functional modules. Enrichment of communities was performed using Reactome, and KEGG pathways and GO terms using g:Profiler [61], as different tools may provide complementary data. The characterization of the communities was based on the Reactome pathway’s enrichment. The counts of cases, variants and genes affected by UR LoF SNVs within the biological communities of the PPI network were also calculated. 

### 2.6. Protein–Protein Interaction Network Gene Validation in Independent Datasets

In this step, we performed a PPI network gene validation in other ASD datasets. We identified the genes of the network that are targeted by UR de novo mutations in the ASD dataset from the SSC “denovo-db version 1.6.1”, available in denovo-db, Seattle, WA, USA (URL: http://denovo-db.gs.washington.edu (accessed on 22 January 2019)). We analyzed the SSC dataset “denovo-db 1.6.1” and selected genes with UR (Exome Aggregation Consortium (EXAC) [62] MAF < 0.001) variants in ASD patients with predicted pathogenic impact in protein structure and/or function according to SIFT [63] or PolyPhen [64]. We also identified the genes of the network that are targeted by putative de novo Copy Number Variants (CNVs) in the AGP dataset. We selected de novo CNVs detected in probands that include NS genes from our PPI network. Additionally, we only selected CNVs that were detected by more than one algorithm, as implemented in [51]. Finally, we also analyzed whether a gene of the network is included in the SFARI list of ASD candidate genes and the strength of the evidence connecting that gene to ASD (https://gene.sfari.org/database/gene-scoring/ (accessed on 13 March 2019)), selecting genes in the strongest SFARI categories 1, 2 and S (Syndromic). SFARI is an ASD dedicated database that incorporates a gene scoring module that establishes a gene rank according to the strength of the evidence associating a given gene to the disease based on the analyses of several studies with ASD patients. The strongest candidate genes (SFARI categories 1 and 2) come from well-defined evidence on human genetic studies. Category 1 considers rigorous statistical comparisons between cases and controls, yielding genome-wide statistical significance with independent replication to be the strongest possible evidence for a gene; in category 2, these criteria are slightly relaxed. Genes predisposed to ASD in the context of a syndromic disorder (e.g., Fragile X Syndrome) are placed in category (S) and, if there is additional evidence implicating them in idiopathic Autism, will also receive a score from 1 to 3, according to the strength of that evidence (1 and 2 are the strongest categories).

### 2.7. Brain Regional Specificity of Gene Expression of Network Communities

The distribution of PPI network genes across the different brain regions was assessed by gene expression enrichment analysis. We used the Bgee database (https://bgee.org/ (accessed on 15 April 2021)) [65] to perform gene expression enrichment analyses of network communities, filtering for the post-embryonic stage in humans. We selected all the anatomical entities from the UBERON ontology of vertebrate anatomical structures [66], which are children of the “brain” term (UBERON:0000955), exclusively related through a “part_of” relationship type. We used all the brain regions in the analysis and selected the parent–child decorrelation type that examines each ontology term in the context of its parent terms, as implemented in the BgeeDB R package (https://bioconductor.org/packages/release/bioc/html/BgeeDB.html (accessed on 15 April 2021)), which is based on the topGO package (https://bioconductor.org/packages/release/bioc/html/topGO.html (accessed on 30 October 2023)). All other parameters were used with default settings. We constructed a gene expression enrichment heatmap and visualized it with the Seaborn (v0.11.2) Python library (https://seaborn.pydata.org/ (accessed on 15 April 2021)) [67]. 

## 3. Results

### 3.1. Generating a List of Neurotransmission and Synaptic ASD Candidate Genes

Using the gene identification strategy defined above, we compiled a list of 1216 NS candidate genes (Supplementary Table S2a) for ASD. The final list of 1216 genes was selected using the GO annotation tools, KEGG and Reactome pathways. This list also includes 253 NS genes identified in the synaptic databases SynSysNet and SynaptomeDB (Supplementary Figure S1b.)

### 3.2. Identification of Ultra-Rare Loss-of-Function SNVs Targeting Neurotransmission and Synaptic Genes in ASD Exomic Datasets

The identification of NS genes targeted by UR SNVs in ASD patients was carried out using WES datasets available through the ASC. We performed a systematic analysis of exome data from 3938 ASD-affected individuals and 1124 unaffected controls (ASC control cohort). This analysis detected 356 UR SNVs in 208 NS genes (17% of genes of the candidate gene list) (Supplementary Table S2b). These variants are present in 446 cases (11% of cases analyzed), and almost all are private, except for twenty-two variants that occur in two cases and two variants that are shared by three cases. The distribution of SNVs by in silico variant classification is shown in Supplementary Figure S2c. Almost half of the UR SNVs identified disrupt protein function by introducing a premature stop codon in the protein sequence (173 variants of stop gain in 356 SNVs; Supplementary Figure S2c). The genes with higher number of variants are the *PDE11A* gene, with eight variants distributed by eleven cases (0.3% of cases), and the *SYTL3* gene, with six variants distributed by nine cases (0.2% of cases) (Supplementary Table S2b).

### 3.3. Construction of the Protein–Protein Interaction Network Spanned by NS Genes Affected in ASD Probands and Network Community Detection

**Figure 1:**
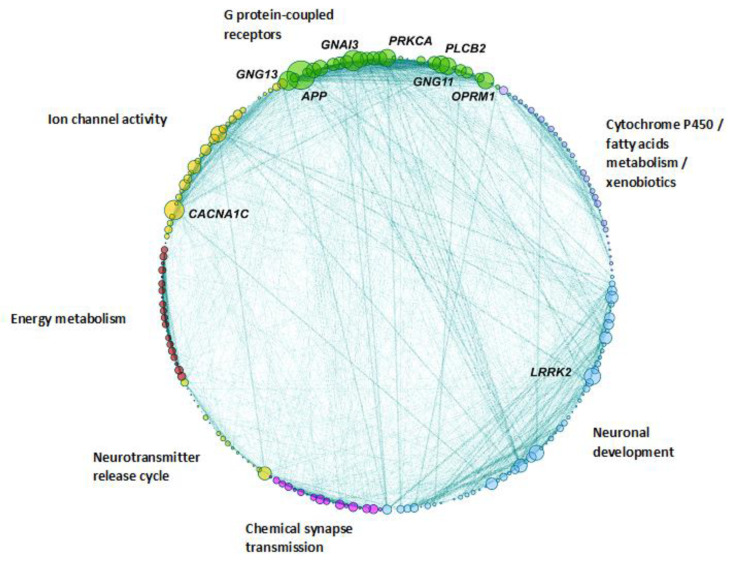
Protein–protein interaction network of NS genes targeted by ultra-rare SNVs. Network nodes represent proteins. Lines represent protein–protein connections. Each functional group (biological community) is highlighted in a different color; the confidence level of the evidence supporting protein–protein connections is proportional to the thickness of the connection line, e.g., thicker connections are better supported than thinner connections. Circle size reflects the node degree, which is the number of connections the node has to other nodes in the entire network. The genes with higher degree levels are indicated next to the correspondent node.

We applied a PPI network analysis considering the genes targeted by predicted UR LoF SNVs. Protein–protein interactions are specific and meaningful from a biological perspective since connected proteins contribute to a shared function while not necessarily physically interacting with each other. The PPI network is composed of 208 NS genes carrying UR SNVs in the 446 ASD cases (Figure 1; Supplementary Tables S3a–c). 

**Figure 2:**
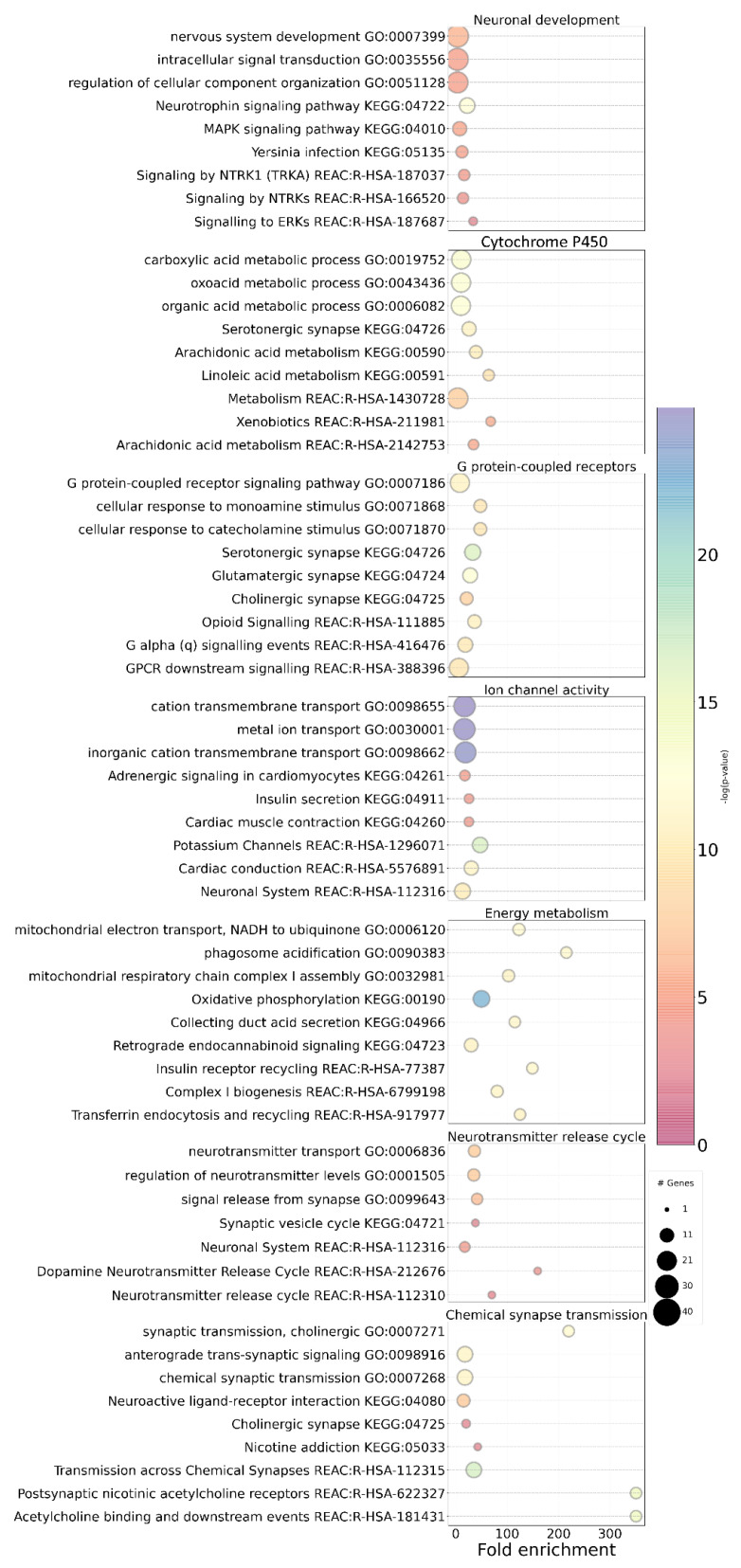
Pathway and GO enrichment for network communities. Circle color represents the magnitude of the fold enrichment, defined as the percentage of genes in our study belonging to a pathway divided by the percentage of genes that are annotated for that pathway; # Genes: Circle size is proportional to the number of network genes implicated in the community.

The Leiden community detection analysis indicates that the network is divided into seven biological communities (Figure 1 and Figure 2; Supplementary Tables S3b and S4a). The seven different communities are indicated with different colors in the network: Ion channel activity community; Chemical synapse transmission community; Energy metabolism community; G protein-coupled receptors community; Metabolism of cytochrome P450, fatty acids and xenobiotics community; Neurotransmitter release cycle community; and Neuronal development community (Figure 1; see also Supplementary Table S4b for a list of the genes present in each community). These biological communities include molecules that intervene in general cellular metabolic and neuronal signaling pathways (Cytochrome P450/fatty acids metabolism/xenobiotics or Ion channel activity communities), those that affect the energetic balance required for the proper functioning of many pleiotropic physiological processes (Energy metabolism community) or those related with neurotransmission pathways (G protein-coupled receptors community, the Chemical synapse transmission community and the Neurotransmitter release cycle community). The network also includes genes that participate directly in neuronal development (the Neuronal development community) and are associated with cell differentiation, neuronal growth, axon guidance and the neuronal immune system.

**Figure 3:**
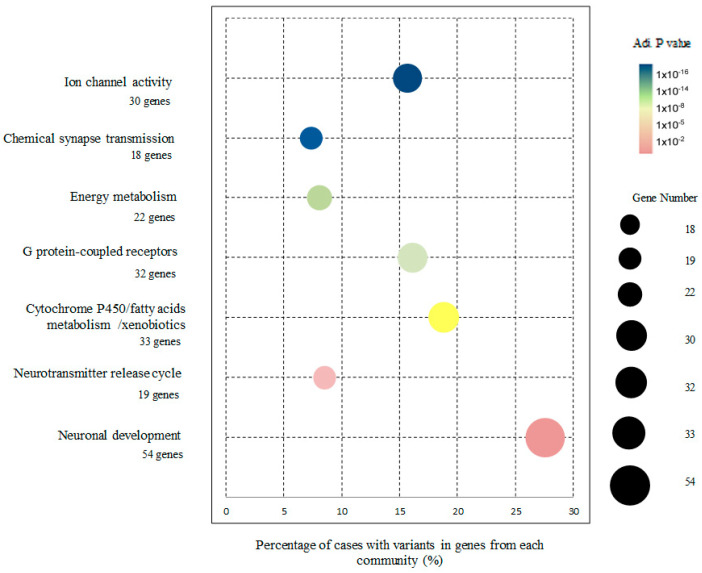
Percentage of cases with variants in genes from each community and the number of genes affected by ultra-rare LoF SNVs within each community, in a total network of 208 genes built from a dataset of 446 cases. Circle size represents the number of network genes implicated in the community and circle color the magnitude of adjusted *p*-value of the enrichment.

The proportion of cases and genes affected by UR LoF SNVs within each of the biological communities of the PPI network is shown in Figure 3 (see also Supplementary Table S3b). The Neuronal development community (27.6% of network cases; 54 genes), the Cytochrome P450/fatty acids metabolism/xenobiotics community (18.8% of network cases; 33 genes) and the G protein-coupled receptors community (16.1% of network cases; 32 genes) are the biological communities with the highest numbers of cases affected by UR SNVs in the analyzed network.

### 3.4. Protein–Protein Interaction Network Gene Validation in Independent Datasets

For validation, we searched for variants in these network genes in large independent datasets, namely, the AGP CNV and the SSC de novo SNV datasets. We also inspected the SFARI candidate gene list (considering only genes with higher evidence (categories 1, 2 or S (Syndromic))) to find additional reports of variants in the network genes. With this search, we identified 34 network genes that were targeted by CNVs or SNVs in previous studies. Sixteen network genes (Supplementary Tables S3b and S3c) showed UR de novo SNVs in the SSC dataset that were classified as deleterious and/or damaging by SIFT/PolyPhen (Supplementary Table S5). In addition, we identified 27 de novo CNVs in the AGP dataset targeting 11 of the network genes (Supplementary Tables S3c and S6). All CNVs were detected by at least two different algorithms. Thirteen of the network genes are also in the SFARI candidate gene list (Supplementary Tables S3b and S3c) in categories 1, 2 or syndromic. The Neuronal development community has the highest number of network genes targeted by de novo CNVs in the AGP dataset (four genes; Supplementary Table S3b). The Ion channel activity community has the highest number of network genes with UR de novo putative pathogenic variants in the SSC dataset (five and nine genes, respectively; Supplementary Table S3b) and the highest number of network genes included in the SFARI high-evidence categories. The genes *ANK2*, *CACNA1C*, *PCDH15*, *SYNE1* and *TRIO* are present in at least one of the datasets and/or in the SFARI gene list. 

### 3.5. Brain Regional Specificity of Gene Expression of Network Communities

**Figure 4:**
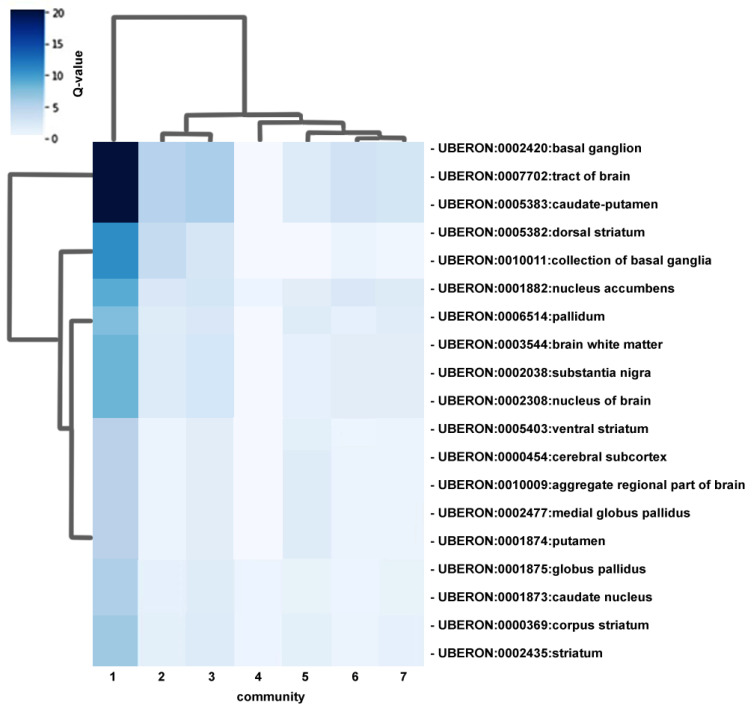
Heatmap representing gene expression enrichment in brain regions of network communities. Each cell (one row and one column) in the heatmap corresponds to a spatial signature: a set of genes from a biological community (communities are represented in the columns) expressed specifically in a brain region (rows). The intensity of the color in each cell (scale in the upper-left side) represents the log-transformed significance of biological community association of that signature. Darker colors in the chart represent higher enrichment probability in the respective brain region. Brain regions are selected from the Uberon database, incorporated in Bgee (https://bgee.org/ (accessed on 15 April 2021)). Bgee expression enrichment analysis was used to calculate *p*-values, which were then converted to Q-values (top-left correspondence to chart colors) to correct for multiple hypothesis testing. The tree branches indicate differences in the degree of gene expression regional specificity between communities. Communities: (1) Neuronal development community; (2) Energy metabolism community; (3) Neurotransmitter release cycle community; (4) Chemical synapse transmission community; (5) Cytochrome P450/fatty acids metabolism/xenobiotics community; (6) G protein-coupled receptors community; (7) Ion channel activity community.

We analyzed whether expression of the network genes in the biological communities is enriched in specific brain regions. The heatmap in Figure 4 shows the enrichment of gene expression in brain regions. Only brain regions for which there is significant enrichment are shown. The Neuronal development community (community 1, Figure 4) is the one with the highest scores of regional specificity of gene expression, as shown by the darker colors representative of higher Q-values and by the tree in the chart, as its branch splits from the branches of the other communities, indicating that the list of genes of this community is more specialized than the others. Considering all the communities together, gene expression in the communities defined in previous steps is mostly shifted towards a set of subcortical structures, many related with the basal ganglia as the globus pallidus, the putamen and the caudate nucleus (dorsal striatum) (Figure 4).

## 4. Discussion

The high heterogeneity of clinical phenotypes in ASD is expected to reflect the diversity of molecular mechanisms and biological processes implicated in this disease and its comorbidities [68]. Hundreds of putative risk genes have been implicated in ASD over time, supporting this hypothesis but hindering the understanding of the disease etiology; nosology; and, most importantly, the definition of pathophysiology and consistent targets for treatment. In this study, we focused on exploring the role of NS genes for their major importance in brain processes that are known to be altered in ASD. However, we wanted to go beyond the identification of ASD risk genes; thus, we sought to clarify the biological processes affected by gene variants and the global networks that link those processes together. This allows for a better depiction of the biological patterns that might be common to a subgroup of patients with similar clinical presentations. Furthermore, it provides a possible explanation for the large number of genes implicated in ASD, as a change in any or several of the multiple genes involved in a network can lead to a similar clinical phenotype within the ASD spectrum. 

Screening a large ASD dataset, we found UR LoF variants in 17% of the selected NS genes, including some interesting novel candidate genes for ASD. The identified SNVs were found in 11% of the individuals analyzed and, as expected since we focused on UR variants, they were almost all private. However, several of the genes were targeted by multiple different variants. For instance, the genes with the highest numbers of variants were *PDE11A* and *SYTL3*. The gene *SYTL3* was not previously associated with ASD, and few studies address the gene *PDE11A* in ASD individuals [69,70]; however, these genes encode molecules that are very important for brain function. The *PDE11A* gene encodes a member of the Phosphodiesterases (PDEs) protein superfamily, which are enzymes that regulate the intracellular levels of cyclic adenosine monophosphate and cyclic guanosine monophosphate, and, consequently, play a central role in multiple cellular functions [71]. Recently, an exome sequencing study revealed *PDE11A* as a novel candidate for Alzheimer’s disease (AD) and found significantly decreased protein levels of *PDE11A* in brain samples of AD patients [72]. Studies on mice indicate that this gene is required for the formation of social interactions [73]. The *PDE11A* gene may also be a target for the development of strategies for personalized medicine, as studies in other neuropsychiatric disorders show that genetic variation in this gene is associated with drug response [74,75]. The second gene with the highest number of variants in our study, *SYTL3*, encodes a protein belonging to a family of peripheral membrane proteins that play a role in vesicular trafficking [76]. Recent studies show that *SYTL3* regulates neuronal migration and neurotransmitter release in human neurons [77]. Whole-genome sequencing studies highlight *SYTL3* as a new candidate gene for AD [78]. 

The NS genes carrying UR LoF variants in the analyzed dataset defined seven biological communities. This network community detection analysis suggests that ASD’s etiology may result from a combination of specific synaptic pathways with other pathways that are more ubiquitous and occur in several organs and systems. For instance, previous studies implicate many of the biological processes underlying the communities identified in this study in ASD, such as ion channels [32,79,80,81]; imbalances in neuronal development and in synaptic processes including synapse formation, stability and plasticity [82]; and in excitatory and inhibitory synapses [83]. Several neurotransmission systems, such as the cholinergic system, acetylcholine receptors and N-methyl-d-aspartate receptors (NMDARs), have also been implicated in ASD. 

However, while synaptic and neurotransmitter-related pathways have been well described in ASD, our study also provides evidence supporting the implication of other pathways not so frequently studied in association with this disorder. For instance, metabolism by cytochromes P450 (CYPs) is one of the communities that we identified, which is relevant since CYPs constitute a superfamily of enzymes involved in the metabolism of several compounds that are key for brain physiology [84]. Brain-expressed CYPs are concentrated near drug targets in specific regions and cell types. The local activity of cytochromes P450 plays an essential role in maintaining the levels of bioactive molecules within normal ranges and modulating the metabolism of endogenous neurochemicals such as neurosteroids, dopamine, serotonin, melatonin and exogenous substances, including psychotropics, drugs and neurotoxins [84,85]. Some genes from the Cytochromes P450/fatty acids metabolism/xenobiotics community were previously identified as strong candidates for ASD, including *ALDH5A1* (SFARI category 1), *PCCA* gene (SFARI category S) and *SLC27A4* (SFARI category 2). The G protein-coupled receptors and Ion channel communities also have NS genes with UR LoF mutations. Among the genes included in the G protein-coupled receptors community, we find several well-established ASD candidate genes, such as *HTR3A*, *DRD3*, *OXTR*, *PRKCA* (all in SFARI category 2) and *TRIO* (SFARI category 1). The Ion channel activity community is also composed by a set of candidate genes relevant for ASD, such as *ANK2* and *CACNA1C* (both in SFARI category 1); *SYNE1* (SFARI category 2S); and *AKAP9, CACNA2D* and *SCN9A* (SFARI category 2).

Energy metabolism is another community enriched in NS genes targeted by mutations in the dataset analyzed. Our results suggest that the dysregulation of mitochondrial biology, particularly the mitochondrial complex I NADH ubiquinone oxidoreductase, may be important for ASD, since genes from the Energy metabolism community, such as *NDUFA5, NDUFV2* or *NDUFB3*, are enriched in the mitochondrial complex 1 biological pathway (Figure 2) and molecular functions (Supplementary Table S4a). Mitochondria perform several functions at the synapse, and synapses that contain mitochondria have been shown to have more vesicles, which are key players for synaptic functions such as the uptake, storage and stimulus-dependent release of neurotransmitters [86] and are associated with increased synaptic activity [87,88]. Previous studies suggest that the expression of mitochondrial complexes is decreased in the brain of children with ASD, eventually contributing to alterations in energetic metabolism and to increased oxidative stress [89,90]. Our analyses suggest that genes from the mitochondrial complex I NADH ubiquinone oxidoreductase may contribute to synaptic dysregulation in ASD. It is well supported in the literature that ASD often co-occurs with mitochondrial dysfunction, which may suggest that a disturbance of mitochondrial energy production is an important underlying pathophysiological mechanism in a subset of patients [91,92,93,94,95,96].

Regional analysis of brain gene expression, for the genes included in the defined biological communities, show that the enrichment of spatial gene expression is stronger in several sub-cortical regions. The results highlight the importance of regions such as the basal ganglia, which includes the globus pallidus and the caudate-putamen (dorsal striatum). The basal ganglia is involved in motor functions and in higher-order cognitive processes, speech, social interactions and repetitive behavior [97], reinforcing the notion that variants in genes expressed in this structure may play a role in ASD. Neuroimaging studies in ASD also validate these findings, as stereotyped behavior has been associated with decreased globus pallidus volume [98]. There is evidence that the basal ganglia plays a role in maintaining an inhibitory balance between cortical and subcortical structures, which is important for motor and cognitive functions. In ASD, this inhibitory balance is disturbed, affecting normal cortical activity. In addition, several studies have reported abnormalities in the cerebellum from subjects with ASD [99]. The Neuronal development community (cluster 1; Figure 4) shows the highest scores of regional specificity of gene expression, suggesting that the pathways involved in this community are the ones with mutations targeting more specialized cellular functions.

The biological communities identified in this study are plausible target pathways for therapeutic development. Pharmacological intervention in ASD is especially challenging due to the considerable variability in its clinical presentation. Currently, there are no drugs approved for treatment of the core symptoms of ASD, and evidence-based pharmacological treatment mostly targets co-occurring symptoms including hyperactivity, inattention, impulsivity or irritability [100]. For instance, risperidone is frequently prescribed for the treatment of irritability symptoms in ASD, and acts through an antagonistic effect on dopamine and serotonin receptors [101]. However, research focused on serotonin and dopamine pathways suggests that these neurotransmitters are also relevant molecular targets for core symptom treatment, namely, to improve repetitive behaviors [102,103]. Our study reinforces several candidate pathways as possible biological targets to treat both core ASD symptoms and associated comorbidities, including ubiquitous pathways involved in neuronal development, neurotransmitter release or synapse transmission, as well as cytochrome P450s and brain mitochondrial metabolism. The integration of genetics with clinical data will be crucial for the clarification of the biological pathways underlying the spectrum of ASD symptoms and respective therapeutic targets [104].

Overall, these results reinforce the role of synaptic and neurotransmitter-related processes in the disorder and support the importance of brain CYPs and brain mitochondrial metabolism in ASD pathophysiology. The results also highlight the role of several subcortical structures in ASD, particularly the basal ganglia.

In conclusion, the present study identified important novel candidate genes and defined new biological pathways for ASD that are not usually addressed in brain genetics studies. These provide further evidence for the role of NS genes and the pathways with which these genes interact in ASD, and suggest targets for future personalized medicinal approaches.

## Data Availability

Data sharing not applicable.

## Supplementary Materials

The following supporting information can be downloaded at: https://www.mdpi.com/article/10.3390/biomedicines11112971/s1, Table S1a: Pipeline for candidate NS gene selection; Table S1b: List of studies analyzed from the Simons Simplex Collection; Figure S1a: Workflow for building and analyzing the protein–protein interaction (PPI) network; Figure S1b: The number of NS genes selected through each of the tools used for ASD candidate gene list selection; Table S2a: List of neurotransmission and synaptic (NS) candidate genes; Table S2b: Ultra-rare loss-of-function Single-Nucleotide Variants in ASD cases from the analyzed ASC cohort; Figure S2c: The distribution of ultra-rare SNVs in NS genes by mutation consequence in the analyzed ASC cohort dataset; Table S3a: Statistics for the network of NS genes targeted by predicted pathogenic ultra-rare variants (MAF < 0.1%); Table S3b: Statistics for the seven network biological communities in the present study and in other studies; Table S3c: Network NS genes with evidence in other studies; Table S4a: GO, KEGG and Reactome pathways enrichment table for the seven communities; Table S4b: The 208 network genes and the respective biological community; Table S5: Ultra-rare de novo SNVs in ASD cases from the SSC cohort classified as deleterious and/or damaging by SIFT/PolyPhen; Table S6: Putative de novo CNVs in ASD cases from the AGP cohort affecting network NS genes.

## References

[1] (2013). Diagnostic and Statistical Manual of Mental Disorders: DSM-5.

[2] Sandin S., Lichtenstein P., Kuja-Halkola R., Larsson H., Hultman C.M., Reichenberg A. (2014). The Familial Risk of Autism. JAMA.

[3] Colvert E., Tick B., McEwen F., Stewart C., Curran S.R., Woodhouse E., Gillan N., Hallett V., Lietz S., Garnett T. (2015). Heritability of Autism Spectrum Disorder in a UK Population-Based Twin Sample. JAMA Psychiatry.

[4] Tick B., Bolton P., Happé F., Rutter M., Rijsdijk F. (2016). Heritability of Autism Spectrum Disorders: A Meta-Analysis of Twin Studies. J. Child Psychol. Psychiatry.

[5] Wigdor E.M., Weiner D.J., Grove J., Fu J.M., Thompson W.K., Carey C.E., Baya N., van der Merwe C., Walters R.K., Satterstrom F.K. (2022). The female protective effect against autism spectrum disorder. Cell Genom..

[6] Genovese A., Butler M.G. (2020). Clinical Assessment, Genetics, and Treatment Approaches in Autism Spectrum Disorder (ASD). Int. J. Mol. Sci..

[7] Autism Spectrum Disorders Working Group of The Psychiatric Genomics Consortium (2017). Meta-Analysis of GWAS of over 16,000 Individuals with Autism Spectrum Disorder Highlights a Novel Locus at 10q24.32 and a Significant Overlap with Schizophrenia. Mol. Autism..

[8] Satterstrom F.K., Kosmicki J.A., Wang J., Breen M.S., De Rubeis S., An J.-Y., Peng M., Collins R., Grove J., Klei L. (2020). Large-Scale Exome Sequencing Study Implicates Both Developmental and Functional Changes in the Neurobiology of Autism. Cell.

[9] Zhou X., Feliciano P., Shu C., Wang T., Astrovskaya I., Hall J.B., Obiajulu J.U., Wright J.R., Murali S.C., Xu S.X. (2022). Integrating de Novo and Inherited Variants in 42,607 Autism Cases Identifies Mutations in New Moderate-Risk Genes. Nat. Genet..

[10] Asif M., Martiniano H.F.M.C.M., Vicente A.M., Couto F.M. (2018). Identifying Disease Genes Using Machine Learning and Gene Functional Similarities, Assessed through Gene Ontology. PLoS ONE.

[11] Vilela J., Asif M., Marques A.R., Santos J.X., Rasga C., Vicente A., Martiniano H. (2023). Biomedical Knowledge Graph Embeddings for Personalized Medicine: Predicting Disease-Gene Associations. Expert Syst..

[12] Asif M., Martiniano H.F.M.C., Lamurias A., Kausar S., Couto F.M. (2023). DGH-GO: Dissecting the Genetic Heterogeneity of Complex Diseases Using Gene Ontology. BMC Bioinform..

[13] Beopoulos A., Géa M., Fasano A., Iris F. (2022). Autism spectrum disorders pathogenesis: Toward a comprehensive model based on neuroanatomic and neurodevelopment considerations. Front Neurosci..

[14] De Rubeis S., He X., Goldberg A.P., Poultney C.S., Samocha K., Cicek A.E., Kou Y., Liu L., Fromer M., Walker S. (2014). Synaptic, Transcriptional and Chromatin Genes Disrupted in Autism. Nature.

[15] Abrahams B.S., Geschwind D.H. (2008). Advances in Autism Genetics: On the Threshold of a New Neurobiology. Nat. Rev. Genet..

[16] Lionel A.C., Vaags A.K., Sato D., Gazzellone M.J., Mitchell E.B., Chen H.Y., Costain G., Walker S., Egger G., Thiruvahindrapuram B. (2013). Rare Exonic Deletions Implicate the Synaptic Organizer Gephyrin (GPHN) in Risk for Autism, Schizophrenia and Seizures. Hum. Mol. Genet..

[17] Leblond C.S., Nava C., Polge A., Gauthier J., Huguet G., Lumbroso S., Giuliano F., Stordeur C., Depienne C., Mouzat K. (2014). Meta-Analysis of SHANK Mutations in Autism Spectrum Disorders: A Gradient of Severity in Cognitive Impairments. PLoS Genet..

[18] Quaak I., Brouns M.R., de Bor M.V. (2013). The Dynamics of Autism Spectrum Disorders: How Neurotoxic Compounds and Neurotransmitters Interact. Int. J. Environ. Res. Public. Health.

[19] Marotta R., Risoleo M.C., Messina G., Parisi L., Carotenuto M., Vetri L., Roccella M. (2020). The Neurochemistry of Autism. Brain Sci..

[20] Jamain S., Quach H., Betancur C., Råstam M., Colineaux C., Gillberg I.C., Soderstrom H., Giros B., Leboyer M., Gillberg C. (2003). Mutations of the X-Linked Genes Encoding Neuroligins NLGN3 and NLGN4 Are Associated with Autism. Nat. Genet..

[21] Zoghbi H.Y. (2003). Postnatal Neurodevelopmental Disorders: Meeting at the Synapse?. Science.

[22] Kaizuka T., Takumi T. (2018). Postsynaptic Density Proteins and Their Involvement in Neurodevelopmental Disorders. J. Biochem..

[23] Van Spronsen M., Hoogenraad C.C. (2010). Synapse Pathology in Psychiatric and Neurologic Disease. Curr. Neurol. Neurosci. Rep..

[24] Kumar A., Sundaram S.K., Sivaswamy L., Behen M.E., Makki M.I., Ager J., Janisse J., Chugani H.T., Chugani D.C. (2010). Alterations in Frontal Lobe Tracts and Corpus Callosum in Young Children with Autism Spectrum Disorder. Cereb. Cortex.

[25] Piton A., Gauthier J., Hamdan F.F., Lafrenière R.G., Yang Y., Henrion E., Laurent S., Noreau A., Thibodeau P., Karemera L. (2011). Systematic Resequencing of X-Chromosome Synaptic Genes in Autism Spectrum Disorder and Schizophrenia. Mol. Psychiatry.

[26] Uzunova G., Pallanti S., Hollander E. (2016). Excitatory/Inhibitory Imbalance in Autism Spectrum Disorders: Implications for Interventions and Therapeutics. World J. Biol. Psychiatry.

[27] Gao R., Penzes P. (2015). Common Mechanisms of Excitatory and Inhibitory Imbalance in Schizophrenia and Autism Spectrum Disorders. Curr. Mol. Med..

[28] Toro R., Konyukh M., Delorme R., Leblond C., Chaste P., Fauchereau F., Coleman M., Leboyer M., Gillberg C., Bourgeron T. (2010). Key Role for Gene Dosage and Synaptic Homeostasis in Autism Spectrum Disorders. Trends Genet..

[29] Penzes P., Buonanno A., Passafaro M., Sala C., Sweet R.A. (2013). Developmental Vulnerability of Synapses and Circuits Associated with Neuropsychiatric Disorders. J. Neurochem..

[30] Hutsler J.J., Zhang H. (2010). Increased Dendritic Spine Densities on Cortical Projection Neurons in Autism Spectrum Disorders. Brain Res..

[31] Chattopadhyaya B., Cristo G.D. (2012). GABAergic Circuit Dysfunctions in Neurodevelopmental Disorders. Front. Psychiatry.

[32] O’Roak B.J., Vives L., Girirajan S., Karakoc E., Krumm N., Coe B.P., Levy R., Ko A., Lee C., Smith J.D. (2012). Sporadic Autism Exomes Reveal a Highly Interconnected Protein Network of de Novo Mutations. Nature.

[33] O’Roak B.J., Stessman H.A., Boyle E.A., Witherspoon K.T., Martin B., Lee C., Vives L., Baker C., Hiatt J.B., Nickerson D.A. (2014). Recurrent de Novo Mutations Implicate Novel Genes Underlying Simplex Autism Risk. Nat. Commun..

[34] Gaugler T., Klei L., Sanders S.J., Bodea C.A., Goldberg A.P., Lee A.B., Mahajan M., Manaa D., Pawitan Y., Reichert J. (2014). Most Genetic Risk for Autism Resides with Common Variation. Nat. Genet..

[35] Grove J., Ripke S., Als T.D., Mattheisen M., Walters R.K., Won H., Pallesen J., Agerbo E., Andreassen O.A., Anney R. (2019). Identification of Common Genetic Risk Variants for Autism Spectrum Disorder. Nat. Genet..

[36] Wilfert A.B., Turner T.N., Murali S.C., Hsieh P., Sulovari A., Wang T., Coe B.P., Guo H., Hoekzema K., Bakken T.E. (2021). Recent Ultra-Rare Inherited Variants Implicate Novel Autism Candidate Risk Genes. Nat. Genet..

[37] Iossifov I., Levy D., Allen J., Ye K., Ronemus M., Lee Y., Yamrom B., Wigler M. (2015). Low Load for Disruptive Mutations in Autism Genes and Their Biased Transmission. Proc. Natl. Acad. Sci. USA.

[38] Krumm N., Turner T.N., Baker C., Vives L., Mohajeri K., Witherspoon K., Raja A., Coe B.P., Stessman H.A., He Z.-X. (2015). Excess of Rare, Inherited Truncating Mutations in Autism. Nat. Genet..

[39] Carbon S., Ireland A., Mungall C.J., Shu S., Marshall B., Lewis S. (2009). AmiGO: Online Access to Ontology and Annotation Data. Bioinformatics.

[40] Kanehisa M., Goto S. (2000). KEGG: Kyoto Encyclopedia of Genes and Genomes. Nucleic Acids Res..

[41] Kanehisa M. (2019). Toward Understanding the Origin and Evolution of Cellular Organisms. Protein Sci. Publ. Protein Soc..

[42] Kanehisa M., Furumichi M., Sato Y., Ishiguro-Watanabe M., Tanabe M. (2021). KEGG: Integrating Viruses and Cellular Organisms. Nucleic Acids Res..

[43] Jassal B., Matthews L., Viteri G., Gong C., Lorente P., Fabregat A., Sidiropoulos K., Cook J., Gillespie M., Haw R. (2020). The Reactome Pathway Knowledgebase. Nucleic Acids Res..

[44] Pirooznia M., Wang T., Avramopoulos D., Valle D., Thomas G., Huganir R.L., Goes F.S., Potash J.B., Zandi P.P. (2012). SynaptomeDB: An Ontology-Based Knowledgebase for Synaptic Genes. Bioinformatics.

[45] Von Eichborn J., Dunkel M., Gohlke B.O., Preissner S.C., Hoffmann M.F., Bauer J.M.J., Armstrong J.D., Schaefer M.H., Andrade-Navarro M.A., Le Novere N. (2013). SynSysNet: Integration of Experimental Data on Synaptic Protein-Protein Interactions with Drug-Target Relations. Nucleic Acids Res..

[46] Banerjee-Basu S., Packer A. (2010). SFARI Gene: An Evolving Database for the Autism Research Community. Dis. Model. Mech..

[47] Buxbaum J.D., Daly M.J., Devlin B., Lehner T., Roeder K., State M.W. (2012). The Autism Sequencing Consortium: Large Scale, High Throughput Sequencing in Autism Spectrum Disorders. Neuron.

[48] Karczewski K.J., Francioli L.C., Tiao G., Cummings B.B., Alföldi J., Wang Q., Collins R.L., Laricchia K.M., Ganna A., Birnbaum D.P. (2020). The Mutational Constraint Spectrum Quantified from Variation in 141,456 Humans. Nature.

[49] Uddin M., Tammimies K., Pellecchia G., Alipanahi B., Hu P., Wang Z., Pinto D., Lau L., Nalpathamkalam T., Marshall C.R. (2014). Brain-Expressed Exons under Purifying Selection Are Enriched for de Novo Mutations in Autism Spectrum Disorder. Nat. Genet..

[50] Alonso-Gonzalez A., Rodriguez-Fontenla C., Carracedo A. (2018). De Novo Mutations (DNMs) in Autism Spectrum Disorder (ASD): Pathway and Network Analysis. Front. Genet..

[51] Pinto D., Pagnamenta A.T., Klei L., Anney R., Merico D., Regan R., Conroy J., Magalhaes T.R., Correia C., Abrahams B.S. (2010). Functional Impact of Global Rare Copy Number Variation in Autism Spectrum Disorders. Nature.

[52] Pinto D., Delaby E., Merico D., Barbosa M., Merikangas A., Klei L., Thiruvahindrapuram B., Xu X., Ziman R., Wang Z. (2014). Convergence of Genes and Cellular Pathways Dysregulated in Autism Spectrum Disorders. Am. J. Hum. Genet..

[53] Szatmari P., Paterson A.D., Zwaigenbaum L., Roberts W., Brian J., Liu X.-Q., Vincent J.B., Skaug J.L., Thompson A.P., Autism Genome Project Consortium (2007). Mapping Autism Risk Loci Using Genetic Linkage and Chromosomal Rearrangements. Nat. Genet..

[54] Anney R., Klei L., Pinto D., Regan R., Conroy J., Magalhaes T.R., Correia C., Abrahams B.S., Sykes N., Pagnamenta A.T. (2010). A Genome-Wide Scan for Common Alleles Affecting Risk for Autism. Hum. Mol. Genet..

[55] Anney R., Klei L., Pinto D., Almeida J., Bacchelli E., Baird G., Bolshakova N., Bölte S., Bolton P.F., Bourgeron T. (2012). Individual Common Variants Exert Weak Effects on the Risk for Autism Spectrum Disorders. Hum. Mol. Genet..

[56] McLaren W., Gil L., Hunt S.E., Riat H.S., Ritchie G.R.S., Thormann A., Flicek P., Cunningham F. (2016). The Ensembl Variant Effect Predictor. Genome Biol..

[57] Szklarczyk D., Gable A.L., Lyon D., Junge A., Wyder S., Huerta-Cepas J., Simonovic M., Doncheva N.T., Morris J.H., Bork P. (2019). STRING V11: Protein–Protein Association Networks with Increased Coverage, Supporting Functional Discovery in Genome-Wide Experimental Datasets. Nucleic Acids Res..

[58] Shannon P., Markiel A., Ozier O., Baliga N.S., Wang J.T., Ramage D., Amin N., Schwikowski B., Ideker T. (2003). Cytoscape: A Software Environment for Integrated Models of Biomolecular Interaction Networks. Genome Res..

[59] Traag V.A., Waltman L., van Eck N.J. (2019). From Louvain to Leiden: Guaranteeing Well-Connected Communities. Sci. Rep..

[60] Rossetti G., Milli L., Cazabet R. (2019). CDLIB: A Python Library to Extract, Compare and Evaluate Communities from Complex Networks. Appl. Netw. Sci..

[61] Raudvere U., Kolberg L., Kuzmin I., Arak T., Adler P., Peterson H., Vilo J. (2019). G:Profiler: A Web Server for Functional Enrichment Analysis and Conversions of Gene Lists (2019 Update). Nucleic Acids Res..

[62] Karczewski K.J., Weisburd B., Thomas B., Solomonson M., Ruderfer D.M., Kavanagh D., Hamamsy T., Lek M., Samocha K.E., Cummings B.B. (2017). The ExAC Browser: Displaying Reference Data Information from over 60,000 Exomes. Nucleic Acids Res..

[63] Ng P.C., Henikoff S. (2003). SIFT: Predicting Amino Acid Changes That Affect Protein Function. Nucleic Acids Res..

[64] Adzhubei I., Jordan D.M., Sunyaev S.R. (2013). Predicting Functional Effect of Human Missense Mutations Using PolyPhen-2. Curr. Protoc. Hum. Genet..

[65] Bastian F.B., Roux J., Niknejad A., Comte A., Fonseca Costa S.S., de Farias T.M., Moretti S., Parmentier G., de Laval V.R., Rosikiewicz M. (2021). The Bgee Suite: Integrated Curated Expression Atlas and Comparative Transcriptomics in Animals. Nucleic Acids Res..

[66] Haendel M.A., Balhoff J.P., Bastian F.B., Blackburn D.C., Blake J.A., Bradford Y., Comte A., Dahdul W.M., Dececchi T.A., Druzinsky R.E. (2014). Unification of Multi-Species Vertebrate Anatomy Ontologies for Comparative Biology in Uberon. J. Biomed. Semant..

[67] Waskom M. (2021). Seaborn: Statistical Data Visualization. J. Open Source Softw..

[68] Vilela J., Martiniano H., Marques A.R., Santos J.X., Rasga C., Oliveira G., Vicente A.M. (2022). Disease Similarity Network Analysis of Autism Spectrum Disorder and Comorbid Brain Disorders. Front. Mol. Neurosci..

[69] Prasad A., Merico D., Thiruvahindrapuram B., Wei J., Lionel A.C., Sato D., Rickaby J., Lu C., Szatmari P., Roberts W. (2012). A Discovery Resource of Rare Copy Number Variations in Individuals with Autism Spectrum Disorder. G3 Genes Genomes Genet..

[70] Yuan H., Dougherty J.D. (2014). Investigation of Maternal Genotype Effects in Autism by Genome-Wide Association. Autism Res. Off. J. Int. Soc. Autism Res..

[71] Levy I., Horvath A., Azevedo M., de Alexandre R.B., Stratakis C.A. (2011). Phosphodiesterase Function and Endocrine Cells: Links to Human Disease and Roles in Tumor Development and Treatment. Curr. Opin. Pharmacol..

[72] Qin W., Zhou A., Zuo X., Jia L., Li F., Wang Q., Li Y., Wei Y., Jin H., Cruchaga C. (2021). Exome Sequencing Revealed PDE11A as a Novel Candidate Gene for Early-Onset Alzheimer’s Disease. Hum. Mol. Genet..

[73] Hegde S., Ji H., Oliver D., Patel N.S., Poupore N., Shtutman M., Kelly M.P. (2016). PDE11A Regulates Social Behaviors and Is a Key Mechanism by Which Social Experience Sculpts the Brain. Neuroscience.

[74] Luo H.-R., Wu G.-S., Dong C., Arcos-Burgos M., Ribeiro L., Licinio J., Wong M.-L. (2009). Association of PDE11A Global Haplotype with Major Depression and Antidepressant Drug Response. Neuropsychiatr. Dis. Treat..

[75] Mertens J., Wang Q.-W., Kim Y., Yu D.X., Pham S., Yang B., Zheng Y., Diffenderfer K.E., Zhang J., Soltani S. (2015). Differential Responses to Lithium in Hyperexcitable Neurons from Patients with Bipolar Disorder. Nature.

[76] Fukuda M., Mikoshiba K. (2001). Synaptotagmin-like Protein 1-3: A Novel Family of C-Terminal-Type Tandem C2 Proteins. Biochem. Biophys. Res. Commun..

[77] Dong X., Yang L., Liu K., Ji X., Tang C., Li W., Ma L., Mei Y., Peng T., Feng B. (2021). Transcriptional Networks Identify Synaptotagmin-like 3 as a Regulator of Cortical Neuronal Migration during Early Neurodevelopment. Cell Rep..

[78] Prokopenko D., Morgan S.L., Mullin K., Hofmann O., Chapman B., Kirchner R., Amberkar S., Wohlers I., Lange C., Alzheimer’s Disease Neuroimaging Initiative (ADNI) (2021). Whole-Genome Sequencing Reveals New Alzheimer’s Disease-Associated Rare Variants in Loci Related to Synaptic Function and Neuronal Development. Alzheimers Dement. J. Alzheimers Assoc..

[79] Sanders S.J., Murtha M.T., Gupta A.R., Murdoch J.D., Raubeson M.J., Willsey A.J., Ercan-Sencicek A.G., DiLullo N.M., Parikshak N.N., Stein J.L. (2012). De Novo Mutations Revealed by Whole-Exome Sequencing Are Strongly Associated with Autism. Nature.

[80] Veeramah K.R., O’Brien J.E., Meisler M.H., Cheng X., Dib-Hajj S.D., Waxman S.G., Talwar D., Girirajan S., Eichler E.E., Restifo L.L. (2012). De Novo Pathogenic SCN8A Mutation Identified by Whole-Genome Sequencing of a Family Quartet Affected by Infantile Epileptic Encephalopathy and SUDEP. Am. J. Hum. Genet..

[81] Schmunk G., Gargus J.J. (2013). Channelopathy Pathogenesis in Autism Spectrum Disorders. Front. Genet..

[82] Lin Y.-C., Frei J.A., Kilander M.B.C., Shen W., Blatt G.J. (2016). A Subset of Autism-Associated Genes Regulate the Structural Stability of Neurons. Front. Cell. Neurosci..

[83] Lee E.-J., Choi S.Y., Kim E. (2015). NMDA Receptor Dysfunction in Autism Spectrum Disorders. Curr. Opin. Pharmacol..

[84] Ferguson C.S., Tyndale R.F. (2011). Cytochromes P450 in the Brain: Emerging Evidence for Biological Significance. Trends Pharmacol. Sci..

[85] Kuban W., Daniel W.A. (2021). Cytochrome P450 Expression and Regulation in the Brain. Drug Metab. Rev..

[86] Rojas-Charry L., Nardi L., Methner A., Schmeisser M.J. (2021). Abnormalities of Synaptic Mitochondria in Autism Spectrum Disorder and Related Neurodevelopmental Disorders. J. Mol. Med..

[87] Smith H.L., Bourne J.N., Cao G., Chirillo M.A., Ostroff L.E., Watson D.J., Harris K.M. (2016). Mitochondrial Support of Persistent Presynaptic Vesicle Mobilization with Age-Dependent Synaptic Growth after LTP. eLife.

[88] Cserép C., Pósfai B., Schwarcz A.D., Dénes Á. (2018). Mitochondrial Ultrastructure Is Coupled to Synaptic Performance at Axonal Release Sites. eNeuro.

[89] Chauhan A., Gu F., Essa M.M., Wegiel J., Kaur K., Brown W.T., Chauhan V. (2011). Brain Region-Specific Deficit in Mitochondrial Electron Transport Chain Complexes in Children with Autism. J. Neurochem..

[90] Anitha A., Nakamura K., Thanseem I., Matsuzaki H., Miyachi T., Tsujii M., Iwata Y., Suzuki K., Sugiyama T., Mori N. (2012). Downregulation of the Expression of Mitochondrial Electron Transport Complex Genes in Autism Brains. Brain Pathol..

[91] Oliveira G., Diogo L., Grazina M., Garcia P., Ataíde A., Marques C., Miguel T., Borges L., Vicente A.M., Oliveira C.R. (2005). Mitochondrial Dysfunction in Autism Spectrum Disorders: A Population-Based Study. Dev. Med. Child Neurol..

[92] Correia C., Coutinho A.M., Diogo L., Grazina M., Marques C., Miguel T., Ataíde A., Almeida J., Borges L., Oliveira C. (2006). Brief Report: High Frequency of Biochemical Markers for Mitochondrial Dysfunction in Autism: No Association with the Mitochondrial Aspartate/Glutamate Carrier SLC25A12 Gene. J. Autism Dev. Disord..

[93] Weissman J.R., Kelley R.I., Bauman M.L., Cohen B.H., Murray K.F., Mitchell R.L., Kern R.L., Natowicz M.R. (2008). Mitochondrial Disease in Autism Spectrum Disorder Patients: A Cohort Analysis. PLoS ONE.

[94] Haas R.H. (2010). Autism and Mitochondrial Disease. Dev. Disabil. Res. Rev..

[95] Frye R.E., Rossignol D.A. (2011). Mitochondrial Dysfunction Can Connect the Diverse Medical Symptoms Associated with Autism Spectrum Disorders. Pediatr. Res..

[96] Goldenthal M.J., Damle S., Sheth S., Shah N., Melvin J., Jethva R., Hardison H., Marks H., Legido A. (2015). Mitochondrial Enzyme Dysfunction in Autism Spectrum Disorders; a Novel Biomarker Revealed from Buccal Swab Analysis. Biomark. Med..

[97] Basal Ganglia and Autism—A Translational Perspective—PubMed. https://pubmed.ncbi.nlm.nih.gov/28730641/.

[98] Estes A., Shaw D.W.W., Sparks B.F., Friedman S., Giedd J.N., Dawson G., Bryan M., Dager S.R. (2011). Basal Ganglia Morphometry and Repetitive Behavior in Young Children with Autism Spectrum Disorder. Autism Res. Off. J. Int. Soc. Autism Res..

[99] Becker E.B.E., Stoodley C.J. (2013). Autism Spectrum Disorder and the Cerebellum. Int. Rev. Neurobiol..

[100] Stepanova E., Dowling S., Phelps M., Findling R.L. (2017). Pharmacotherapy of emotional and behavioral symptoms associated with autism spectrum disorder in children and adolescents. Dialogues Clin. Neurosci..

[101] Mano-Sousa B.J., Pedrosa A.M., Alves B.C., Galduróz J.C.F., Belo V.S., Chaves V.E., Duarte-Almeida J.M. (2021). Effects of Risperidone in Autistic Children and Young Adults: A Systematic Review and Meta-Analysis. Curr. Neuropharmacol..

[102] Hollander E., Soorya L., Chaplin W., Anagnostou E., Taylor B.P., Ferretti C.J., Wasserman S., Swanson E., Settipani C. (2012). A double-blind placebo-controlled trial of fluoxetine for repetitive behaviors and global severity in adult autism spectrum disorders. Am. J. Psychiatry.

[103] Mandic-Maravic V., Grujicic R., Milutinovic L., Munjiza-Jovanovic A., Pejovic-Milovancevic M. (2022). Dopamine in Autism Spectrum Disorders-Focus on D2/D3 Partial Agonists and Their Possible Use in Treatment. Front. Psychiatry.

[104] More R.P., Warrier V., Brunel H., Buckingham C., Smith P., Allison C., Holt R., Bradshaw C.R., Baron-Cohen S. (2023). Identifying rare genetic variants in 21 highly multiplex autism families: The role of diagnosis and autistic traits. Mol. Psychiatry.

